# Chemical strategies for strand selection in short-interfering RNAs

**DOI:** 10.1039/d0ra07747j

**Published:** 2021-01-11

**Authors:** Andrew J. Varley, Jean-Paul Desaulniers

**Affiliations:** Faculty of Science, University of Ontario Institute of Technology Oshawa Ontario L1G 0C5 Canada jean-paul.desaulniers@ontariotechu.ca +1 905 721 3304 +1 905 721 8668 (ext. 3621)

## Abstract

Therapeutic small interfering RNAs (siRNAs) are double stranded RNAs capable of potent and specific gene silencing through activation of the RNA interference (RNAi) pathway. The potential of siRNA drugs has recently been highlighted by the approval of multiple siRNA therapeutics. These successes relied heavily on chemically modified nucleic acids and their impact on stability, delivery, potency, and off-target effects. Despite remarkable progress, clinical trials still face failure due to off-target effects such as off-target gene dysregulation. Each siRNA strand can downregulate numerous gene targets while also contributing towards saturation of the RNAi machinery, leading to the upregulation of miRNA-repressed genes. Eliminating sense strand uptake effectively reduces off-target gene silencing and helps limit the disruption to endogenous regulatory mechanisms. Therefore, our understanding of strand selection has a direct impact on the success of future siRNA therapeutics. In this review, the approaches used to improve strand uptake are discussed and effective methods are summarized.

## Abstract 2

Therapeutic small interfering RNAs (siRNAs) are double stranded RNAs capable of potent and specific gene silencing through activation of the RNA interference (RNAi) pathway.

## Graphical abstract

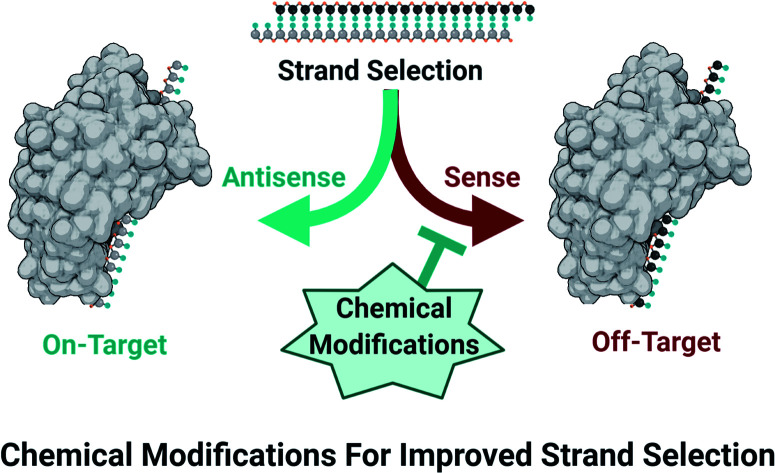

## Introduction

With the approval of the first two siRNA drugs in 2018 and 2019, small interfering RNA (siRNA) therapeutics have gained new enthusiasm. These class of drugs target RNA within the cytoplasm for the potent silencing of nearly any gene transcript or viral RNA genome. Along with other oligonucleotide therapeutics, siRNA are categorized as informational drugs, such that the gene target (pharmacophore) can be designed separately from the chemical modifications used to improve stability, delivery, and specificity of the drug.^1^ Unlike traditional small molecule drugs, this allows the development of chemical modifications that can be broadly applied for many siRNA therapeutics and affords the rapid development against new targets. Fittingly, the recent pandemic caused by the severe acute respiratory syndrome coronavirus 2 (SARS-CoV-2) has led to several siRNA drug candidates in clinical trials to combat the virus.^2^ The future of siRNA therapeutics is therefore a promising option for both endogenous gene silencing and combating novel viral outbreaks.

**Fig. 1:**
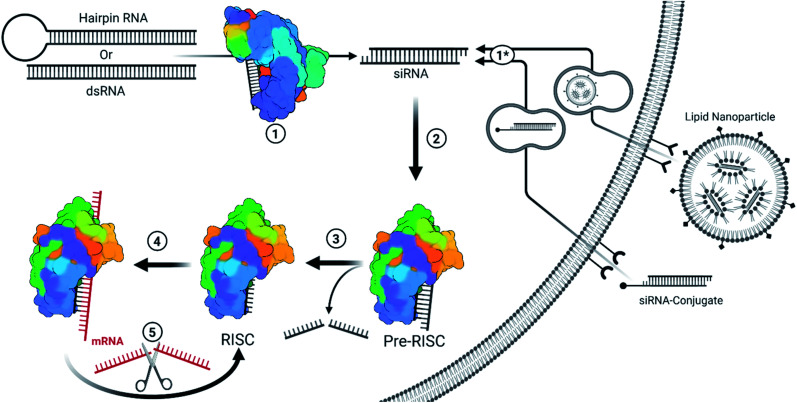
Simplified overview for the endogenous/viral and therapeutic siRNA pathway.^3^ (1) Fully complementary endogenous hairpin RNA or double stranded RNA (dsRNA) within the cytoplasm are recognized and cleaved into ∼19 bp long RNA with an additional two nucleotide-long overhang on each end. These siRNAs can be of endogenous or viral origin.^4^ (1*) Therapeutic siRNAs are typically designed or formulated to bind to a cell surface receptor where they gain entry *via* the endocytic pathway. Mechanisms behind the timing and release of siRNA-conjugates are not well understood but they require extensive backbone modifications to resist degradation within endosomes. Depending on the design, the conjugate may be removed once inside the cell. Givosiran, lumasiran, and inclisiran (which is approved in Europe and has been submitted for FDA approval) utilize a trivalent GalNAc conjugate for cell entry. Lipid nanoparticles provide a protective barrier for siRNA and contain ionizable lipids that become positively charged in the presence of the acidic endosomes, resulting in the destabilization of the anionic endosomal membrane and release of siRNA.^5^ Patisiran is delivered *via* lipid nanoparticle. Since therapeutic siRNAs are designed to match Dicer products, they bypass step 1. (2) The distinct siRNA structure is recognized by the pre-RNA induced silencing complex (pre-RISC) and oriented such that the guide strand (ideally the antisense strand) is bound by AGO2. Most chemical modifications that impact strand selection do so at this step. Recognition depends on a 5′ phosphate on the guide strand, whose phosphorylation status is kept in balance through the action of phosphatase(s) and Clp1 kinase. Modifications at the 5′ end can inhibit phosphorylation by Clp1 kinase, an obstacle which can be overcome with stabilized phosphate mimic modifications. (3) The passenger strand is sliced and released from AGO2 to form the RISC. An alternative pathway allows single stranded siRNA (ss-siRNA; not depicted) with a 5′ phosphate to be recruited by AGO2; however, ss-siRNA are not a substrate for biological phosphorylation and require a phosphate or stabilized phosphate mimic to be present prior to treatment. Some modifications can interrupt passenger strand cleavage, preventing maturation of the RISC with the captured strand. (4) The mature RISC will search for mRNA sequences that are complimentary to the guide strand sequence. The seed sequence (bases 2–8) act as a primer for hybridization of the rest of the strand. Seed region binding can lead to off-target effects, particularly in the 3′ untranslated region of genes. Once the seed region is bound, hybridization with complementary sequences propagates down the strand. (5) Once completely hybridized with a complementary mRNA sequence, the mRNA is cleaved and released in a similar manner as a passenger strand. The RISC is then free to repeat this process to target mRNA in a catalytic fashion.

siRNA therapeutics are capable of rapid, efficient, and selective gene silencing with a single treatment lasting many months. Therapeutically, this occurs through the delivery of chemically modified, 19–21 nucleotide long, double-stranded RNAs which carry two nucleotide-long 3′ overhangs. Strands destined for the siRNA pathway typically exhibit perfect base pairing, while imperfectly paired strands lead to miRNA regulation. The primary difference between the two pathways are the mechanisms for gene silencing: siRNAs are typically loaded into the RNA guided nuclease Argonaute-2 (AGO2) which enzymatically cleaves complementary mRNA targets; while miRNAs typically inhibit protein translation by guiding Argonaute family proteins to bind the 3′ untranslated region (3′UTR) of genes.^6^ A brief description of siRNA mechanisms, and delivery methods for currently FDA-approved siRNAs, are described in Fig. 1.

An siRNA molecule is recognized by cytosolic Argonaute 2 (AGO2), forming the pre-RNA induced silencing complex (pre-RISC). AGO2 preferentially orients the dsRNA in the pre-RISC based on the properties of the sequence, thermodynamic stability, and structure of the 5′ ends.^7,8^ This orientation determines which strand of the siRNA duplex is chosen as the guide strand, and ideally selects the antisense strand (complementary to the target sequence). The sense strand is then oriented as the passenger strand and degraded to form the RISC. The RISC then searches for perfectly complementary mRNA sequences for cleavage; however, microarray datasets have revealed that partial recognition within the seed region (nucleotides 2–8) to the 3′UTR of mRNAs leads to widespread gene down regulation in an miRNA-like fashion.^9–12^ Mitigating these off-target effects is possible through chemical modification and sequence design strategies which reduce seed-region binding and possible matches in the 3′UTR.^13–17^ Importantly, each siRNA strand carries the potential to exhibit a range of distinct gene silencing effects.^18^ Therefore, limiting sense strand selection plays a critical role in managing these off-target effects.

**Fig. 2:**
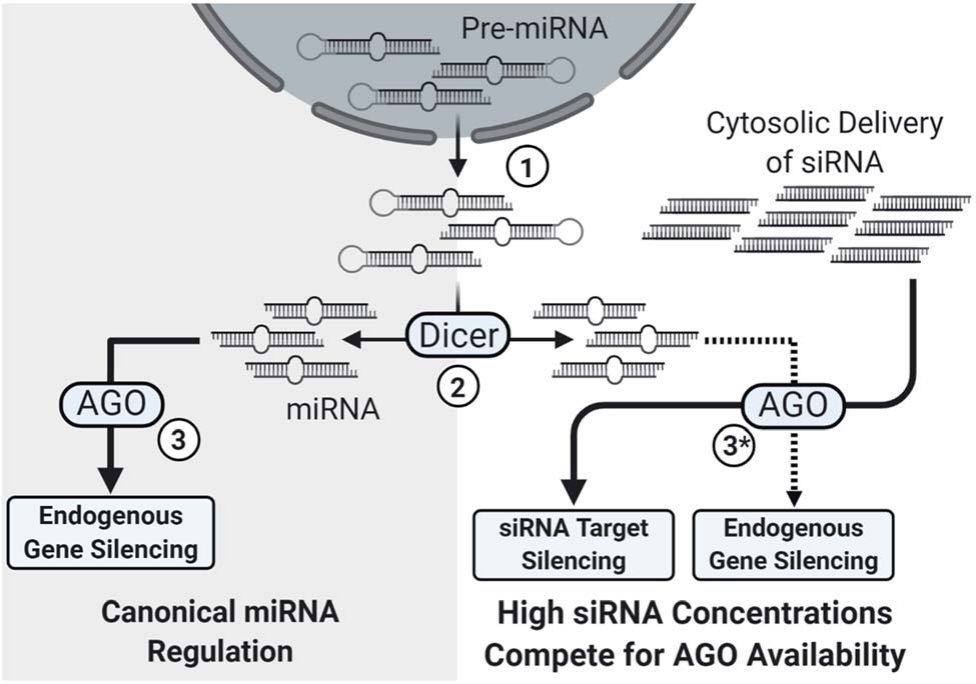
Simplified overview of the impact of high siRNA delivery on miRNA regulation. (1) Pre-miRNAs, processed within the nucleus, are exported to the cytosol. (2) Pre-miRNAs are processed into their characteristic length and structure by Dicer into miRNAs. (3) miRNAs typically depend on the nuclease inactive Argonaute family proteins (AGO1, AGO3, AGO4) for target gene silencing; however, AGO2 also catalyzes several miRNA-guided mRNA cleavages.^27^ Notably, AGO2 is essential for murine viability^28^ and some murine miRNAs require AGO2, instead of Dicer, for maturation.^29,30^ (3*) High cytosolic load of siRNAs saturates the silencing machinery which limits miRNA processing, uptake, and activity, leading to broad de-repression of miRNA regulated genes.^19^

Another concern with sense strand uptake is the saturation of RNAi machinery as siRNAs compete with the endogenous miRNA pool. If the antisense strand has poor activity or the sense strand is selected by the RISC, a higher concentration of siRNAs is required to achieve potent gene silencing. The increased siRNA load poses a higher risk for saturating the RNAi machinery, preventing endogenous miRNA activity and upregulation of miRNA-controlled genes (Fig. 2).^19^ An estimated 10^3^ to 10^4^ RISC molecules are present in the average mammalian cell, corresponding to an intracellular concentration of about 3 to 5 nM.^20^ Saturation will occur once the number of cytoplasmic siRNAs approach comparable numbers; however, effective cell uptake methods rely on endocytosis and less than 0.01% of administered siRNAs may enter into the cytoplasmic pool.^21–23^ An advantage of endocytosis is that an endosomal reservoir for siRNAs facilitates the slow and prolonged release of siRNAs, thus preventing saturation while extending action of the drug. Indeed, months-long duration has been reported in the highly stable GalNAc_3_ conjugated siRNAs which enter cells through recognition of the conjugated GalNAC_3_ by the hepatic asialoglycoprotein receptor.^21,24–26^ Preventing saturation of siRNA machinery is therefore a combination of cytoplasmic availability, potency of the antisense strand, and preventing sense strand selection.

Delivery of siRNA therapeutics continues to be a formidable challenge for wider application and success. The size and charge of siRNAs prevents passive entry into cells and leads to clearance *via* liver accumulation (secreted into the gallbladder and emptied into the intestine) and renal filtration.^31,32^ To prevent the rapid degradation by blood serum nucleases, siRNAs must either be extensively modified or encapsulated in a protective carrier molecule such as a lipid nanoparticle (LNP). Formulation of the LNPs facilitate delivery of the molecules to tissues such as the liver. For siRNAs that lack a carrier (naked siRNAs), cellular delivery is achieved through chemical conjugation to biologicals. Delivery systems have therefore been pivotal to enable cellular entry of siRNA.^33,34^

Nucleic acid modifications provide indispensable nuclease resistance and delivery characteristics to siRNAs; however, they can also provide a means to control immune activation, reduce miRNA-like off target effects, and reduce sense strand uptake. The number of chemically modified nucleic acids, mimics, and linkers developed to date is vast and the effects vary based on both their structure and location within an siRNA molecule. Recent reviews have brilliantly discussed the state of the art of siRNA therapeutics;^35,36^ however, a comprehensive review detailing the chemical modifications used for controlling strand selection is absent. Therefore, this review will focus on chemical modifications that impact the strand selection process and summarizes successful strategies.

## Nomenclature

Throughout several publications, the terms guide and passenger strand are often used interchangeably with antisense and sense strand, respectively, yet the distinction between terms is particularly important when considering strand selection. The strand carrying the complementary sequence to the target is the antisense strand and, if properly designed, will be preferentially chosen to be the guide strand. In this case, the passenger strand is the sense strand. However, one cannot state that a strand is the guide strand until the 5′ end of the strand has been captured within the MID domain of AGO2. Accordingly, the sense strand can be selected as the guide strand, resulting in an antisense passenger strand. Authors and readers must be cautious when using guide and passenger strand terminology.

This review extensively discusses nucleotide locations within stands and simplifies their description using the nomenclature as [AS/SS:##] where AS is the antisense strand, SS is the sense strand, and ## is the nucleotide number from the 5′ end of the corresponding strand. For example, SS:14 refers to the 14^th^ nucleotide from the 5′ end of the sense strand.

Lastly, authors often discuss modifications as tolerated or not tolerated; however, it is important to recognize whether this is in context of the antisense strand or entire siRNA. If a modification is not tolerated in the antisense strand, it may be of use in the sense strand to prevent uptake. Whereas a modification that is not tolerated within siRNA will exhibit poor RNAi gene silencing regardless of which strand it is placed.

## 5′ Phosphate/carbon modifications

The 5′ phosphate is arguably the most important region when considering uptake by AGO2. A phosphate group at the 5′ end of a strand is essential for AGO2 uptake.^37,38^ Within the cell, siRNAs are recognized by Clp1 (kinase) and phosphatase(s) which maintain a balance of 5′ phosphorylated and free hydroxyl ends.^39,40^ Modification of the 5′ end can impact the efficiency of these enzymes, resulting in a shift in the phosphorylation status and therefore likelihood for uptake of the associated strand.

The development of an effective stabilized 5′ phosphate mimic must facilitate RNAi activity by overcoming three primary challenges. They must (1) sterically fit within the deep binding pocket of AGO2; (2) permit interactions with AGO2 side chains without significant distortion to the attached nucleoside; and (3) prevent hydrolysis by phosphatases within the cell. Such strict requirements have led to relatively few effective 5′ phosphate mimics.

### Methylene- and vinyl-phosphonate

Stabilized 5′ phosphate mimics are best suited for antisense strands that carry modifications within the first three nucleotides of the 5′ end. These modifications can reduce Clp1 kinase activity, thereby preventing intracellular phosphorylation of the strand.^41,42^ Furthermore, the synthetic addition of a natural phosphate moiety is not an effective solution as these modifications do not simultaneously prevent hydrolysis of the terminal phosphate. The resulting shift in phosphorylation status is of considerable concern as current siRNA therapeutic design pushes towards complete strand modification to improve tissue retention and resist degradation.^43,44^ Importantly, an effective mimic is also advantageous for single stranded siRNAs (ss-siRNAs), which consists solely of an antisense strand that is recruited by AGO2, as they are poorly phosphorylated in the cell.^45–47^

**Fig. 3:**
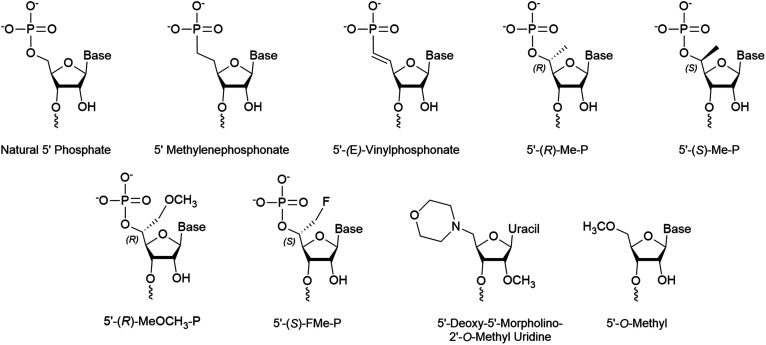
Chemical modifications of the 5′ terminus of siRNA that impact strand selection and/or antisense activity.

The 5’-(*E*)-vinylphosphonate (5′-*E*-VP) mimic (Fig. 3), which contains a *trans* double bond between the 5′ and 6′ carbons, is a well-studied and promising phosphate mimic. Although the 5′-*E*-VP has been used in enzyme kinetics studies since 1976,^48^ its role in RNAi is relatively recent. In a screen of 5′ methylenephosphonate (Fig. 3) analogues for siRNA and ss-siRNA activity, those with the 5′-*E*-VP modification were the most promising.^47^ It was further analyzed in an extensive 5′ phosphate analogue screen for ss-siRNA and siRNA which established stereo-electronic requirements for 5′-phosphate analogues.^45^ This study identified several modifications carrying a 5′ phosphate analogue for antisense tolerance in both ss-siRNA and siRNA including the 5′-*E*-VP.

The addition of a 5′-*E*-VP to GalNAc_3_ conjugated siRNA targeting ApoB improved the IC_50_ by 3 to 20 fold in mouse hepatocytes.^49^ Recall that conjugated siRNAs are delivered nakedly and must be heavily modified to resist degradation. Since these strands are more readily recognized by phosphatases than kinases, a non-cleavable 5′-*E*-VP circumvents this issue. In a similar study targeting mouse PTEN and human ApoC3 in transgenic mouse models, the addition of the 5′-*E*-VP dramatically improved the IC_50_ and/or ED_50_.^50^ For siRNA carrying hydrophobic conjugates, the addition of the 5′-E-VP improved tissue retention, silencing activity, and duration of effect.^51^

Compared to an unmodified 5′ phosphate, the 5′-*E*-VP improved binding affinity of AGO2 by approximately 2-fold.^52^ Notably, the addition of internal 2′-F and 2′-*O*-Me sugar modifications additionally increased binding affinity by approximately 25%. The alignment and position of the 5′-*E*-VP superimposes almost perfectly with a similar complex which contains a natural 5′ phosphate. The modification results in a slight rotational shift that does not substantially alter the distance between AGO2 side chains and the phosphate moiety. Furthermore, the double bond present in the 5′-*E*-VP results in a 1 Å shift in the position of the sugar and brings the 5′ nucleobase 0.7 Å closer to the base-specificity loop G524-T526. Presumably, this may result in a stronger preference for A or U by the 5′ AGO2 pocket, improving strand uptake. The similar size, conservation of AGO2 sidechain interactions, phosphatase resistance, and resulting efficiency of siRNA carrying a 5′-*E*-VP provide a unique advantage for several siRNAs with 5′ end modifications.

### 5′ Hydrogen substitutions

Several 5′ terminal modifications which focus on the substitution at the 5′ carbon have been investigated in siRNA and ss-siRNA. The 5′-(*R*)-Me-P (Fig. 3) modification comes from the addition of a methyl group with *R* stereochemistry at the 5′ carbon. With siRNAs or ss-siRNAs, this modification improved the IC_50_ by approximately 2 or 3-fold, respectively.^45^ Of note, the 5′-(*S*)-Me-P (Fig. 3) stereoisomer had no impact on the IC_50_ of siRNA. The authors theorized that the *R* stereochemistry organizes conformational preference for the 5′ phosphate in AGO2-RNA complexes.^53^ The 5′-(*R*)-MeOCH_2_-P (Fig. 3) modification improved siRNA and ss-siRNA activity by 2 and 20-fold, respectively.^45^ Notably, the increased steric bulk at the 5′ carbon does not seem to interfere with recognition by AGO2. The 5′-(*S*)-FMe-P (Fig. 3) improved the IC_50_ of siRNA and ss-siRNA by approximately 2-fold.^45^ To their surprise, a mono- or di-fluorination of the 5′ carbon only marginally improved or decreased activity, respectively, suggesting that more than electronic factors likely play a role. While this study identified new modifications that enhance antisense strand activity, the 5′-(*E*)-VP phosphate mimic is the preferred choice for phosphate stabilization.

### 5′ Alternative structures

Any modification to the 5′ phosphate that doesn't closely mimic a phosphate group is likely to disrupt activity of the corresponding strand, which was exemplified by the poor activity of an siRNA with a 5′-*O*-methyl (Fig. 3).^37^ This prevents the necessary phosphorylation that is required for strand activity. In contrast, modifications at the 5′ terminal on the antisense strand must be intracellularly removed unless they allow phosphorylation or closely resemble a phosphate.

In a recent screen, a 5′-deoxy-5′-morpholino-2′-*O*-methyl uridine (Fig. 3) on the sense strand reduced its activity by over 3-fold.^54^ This also resulted in approximately a 0.7-fold increase of antisense strand activity which was attributed to low sense strand uptake by AGO2. Molecular modeling of the 5′ phosphate region predicted the loss of all but a single hydrogen bond between Lys-570 and the morpholino ring oxygen. Additionally, the authors suggest that repulsive interactions between the cationic morpholino and residues Lys-570 and Lys-533 may occur. Their study also identified that an inverted abasic site and 5′-deoxy-2′-*O*-methyl uridine also displayed preferable, yet less pronounced, strand activities. Therefore, the morpholino modification may be more effective than a 5′-*O*-methyl for preventing sense strand uptake.

Conjugates are typically added to the 5′ or 3′ end of an siRNA or dsRNA molecule;^26,55–59^ however, success has also been achieved using internally conjugated strands.^60,61^ Internal modifications leave the ends free for other modifications and the location of the modifications can have a large impact on activity and/or strand selection. When considering conjugates which are linked through a phosphate intermediate, it is important to determine the conjugate's stability against enzymatic removal/cleavage. If cleavage occurs in blood serum, the siRNA will be unable to enter the desired cells, while intracellular removal of the conjugate may improve or worsen strand selection.

Early studies suggest that the 5′ antisense strand end will not tolerate conjugation,^62–64^ supported by the identification of a deep 5′ binding pocket in the MID domain of AGO2.^65,66^ Some studies do indicate that 5′ conjugates are tolerated on the antisense strand;^55,67^ however, they were connected using a phosphodiester bond and its intracellular stability was not determined. Therefore, the activity seen may be the cleavage product of the conjugated siRNA. Fittingly, strategies that rely on the removal of a conjugate have been successful. One method is through the use of longer dsRNA to recruit Dicer and cleave the conjugate end.^68,69^ This approach limits the number of nuclease-resistant chemical modifications that can be placed in the strand. Photolabile,^70^ acid labile, and reducible disulfide linkages^71^ are additional strategies which have been used to cleave intracellularly undesirable components.

Unsurprisingly, the inhibitory effect that conjugates can have on their corresponding strand has led to most conjugates having been developed on the sense strand. The FDA approved conjugate siRNA, Givosiran, carries the multivalent GalNAc_3_ cluster on the 3′ end of the sense strand. Generally, most conjugates will decrease activity of the strand to which it is bound; however, the nature of the conjugate could also play a role. For more detail on conjugates currently in development, a recent review provides a detailed list of conjugates and their function.^71^

## Internal modifications

### Phosphate backbone modifications

Numerous phosphate backbone modifications have been developed, yet few have been investigated for their role in strand selection. Strand selection depends heavily on backbone interactions with AGO2 and therefore stereospecificity of modification also plays a role. A recent patent identifies the impact of stereospecific phosphorothioates on strand selection where the *S* and *R* configuration are preferred at the 3′ and 5′ ends of the antisense strand, respectively.^72^ By assumption, the opposite chirality on the sense strand may improve its passenger strand quality.

**Fig. 4:**
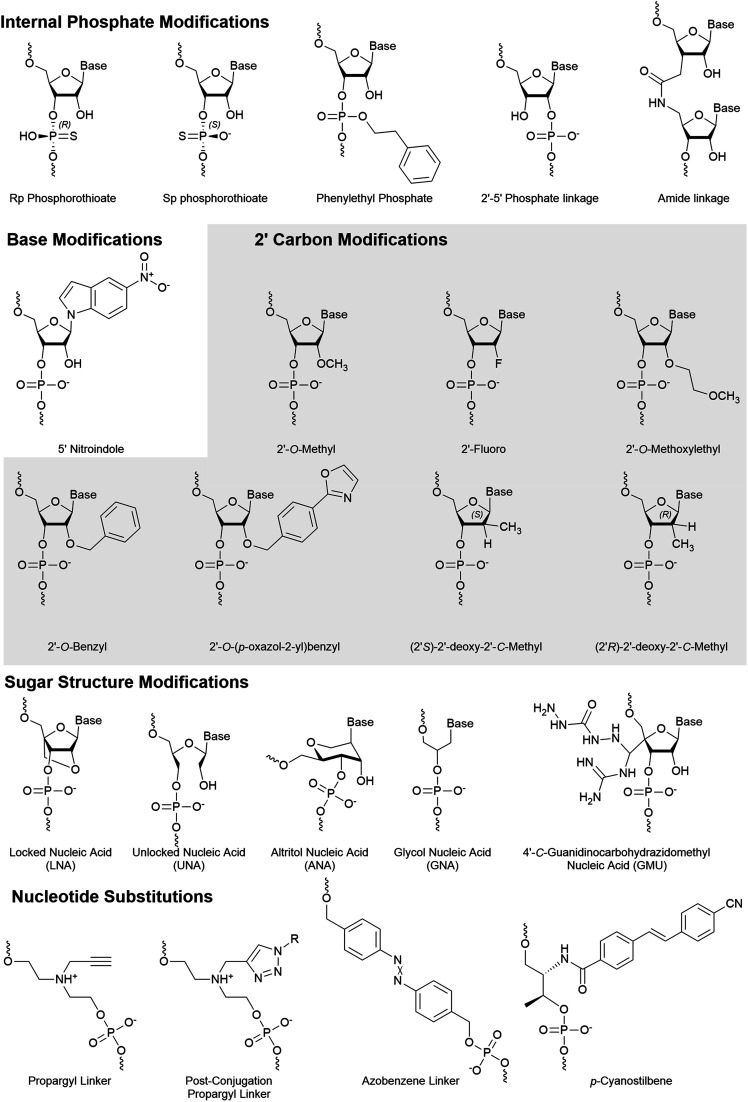
Internal chemical modifications to siRNA that impact strand selection.

An amide linker (Fig. 4) replaces the 3′–5′ phosphate link with a neutral, nuclease resistant linker.^73,74^ The amide linker is surprisingly well tolerated at most positions of the antisense strand, while the positions AS:1, AS:2, AS:3, AS:14, or AS:15 did not tolerate the modification well and the extent of its impact was sequence dependent.^75^ To explore whether this could be exploited for strand selection, the linker was placed at several positions of the sense strand.^76^ When placed between SS:1 and SS:2, sense strand activity was nearly or completely abolished. As seen with other 5′ end modifications, this may be due to poor recognition by Clp1, resulting in the poor phosphorylation status of the strand.^42^

Shifting the location of the phosphate internucleotide linkage with 2′–5′ linkages (Fig. 4) is well tolerated in the sense strand.^77^ Some 2′–5′ phosphate linkages are tolerated on antisense strand, but modification of all six nucleotides from AS:1 to AS:6 reduced activity nearly 8-fold. Positional screening revealed that none of the first six linkages played a substantially larger role than the others and that up to two modifications are relatively well tolerated. Importantly, modification of all siRNA termini led to a significant decrease in target knockdown. Additionally, stability against serum nucleases was improved but not as effectively as phosphorothioate modified siRNA. Strand uptake analysis was only performed on siRNA with 2′–5′ phosphate linkages in the first 2 or 7 linkages of the antisense strand which showed minimal impact on selection. Another recent modification to undergo strand-specific testing is the neutral phenylethyl phosphate (Fig. 4) modification which modestly improves asymmetrical strand activity when simultaneously placed at position SS:2 and SS:15.^78^

### Base modifications

Base modifications are likely to dramatically impact the hydrogen bonding that leads to the thermodynamic stability of dsRNA. Based on the canonical thermodynamic sensory mechanisms, modifications that decrease or increase thermodynamic stability are well suited for the 5′ end of the antisense or sense strand, respectively. While the thermodynamic stability of ends has traditionally been considered the primary factor for strand selection of unmodified siRNAs, a recent study reports that the 5′ terminal base identity plays a larger role in strand selection.^7^ However, while thermodynamic stability and base identity aids in strand selection, chemical modifications are necessary for the abolishment of sense strand activity.

Zhang and colleagues investigated strand activity when a single 5-nitroindole modification (Fig. 4), which acts as a universal base, was placed at each nucleotide of the sense strand.^79^ They analyzed two unique siRNA sequences and found that sense strand activity was reduced, and antisense activity was improved, when the modification was near the 3′ end. While multiple positions provided benefit, modification of AS:15 and SS:15 was further investigated using eight gene targets. In every case, modification of the antisense dramatically reduced antisense knockdown, while modification of the sense strand either improved or had no effect on antisense knockdown. The 5-nitroindole is appealing as it can be introduced in addition to most siRNA designs, which primarily rely on sugar and/or phosphate modifications.

### 2′ Ribose modifications

Numerous modifications have been developed through the modification of the 2′ hydroxyl of the ribose sugar. Replacement of the 2′ hydroxyl generally improves the chemical stability of RNA and often improves nuclease resistance. In addition, many modifications also utilize the electronic interactions that preferentially arrange sugar conformations that impact the thermodynamic stability of strands.

The 2′-*O*-Bn (Fig. 4) modification was characterized at each position of the antisense strand.^80^ When placed at the AS:1 or AS:2 position, targeted mRNA degradation was decreased by approximately 4-fold while modification at AS:14 and AS:16 reduced activity by almost 3-fold. Surprisingly, a spatially more demanding 2′-*O*-(4-oxazol-2-yl)benzyl (Bn4OxAz) (Fig. 4) modification was well tolerated at AS:1 but it reduced activity by up to 16-fold when placed at AS:2, AS:12, AS:13, or AS14. Presumably, placing a 2′-*O*-Bn4OxAz at these positions of the sense strand may be an effective, yet untested, strategy for reducing sense strand uptake.

A single 2′-*O*-Me (Fig. 4) at AS:14, but not AS:13 or AS:15, was found to dramatically reduce RNAi activity in a series of siRNAs.^81^ Quantification of AGO2 loaded strands identified that reduced activity was the result of poor antisense loading. The 2′-OH at AS:14 interacts with a highly conserved sequence across the Argonaute family proteins and modification of AS:14 for miRNA, which works through translational repression, provided similar results. Furthermore, modification of position 14 with a 2′-*O*-MOE was found to have a similar impact on activity while a 2′-F (Fig. 4) was far more tolerated. Recently, a 3′ terminal 2′-*O*-Me was found to be detrimental for 20 nt long siRNA sequences due to PAZ domain interactions but the addition of an extra 2′-F in the seed region can partially recover lost activity.^82^ The 2′-*O*-Me is a widely used modification and several exist in the FDA-approved siRNAs. Notably, both drugs include the modification at SS:14.

2′-*O*-MOE (Fig. 4) modifications are tolerated at several positions; however, are limited due to their larger size which can lead to steric clashes at some positions of the antisense strand.^83^ Interestingly, when placed at position 9 or 10, the 2′-*O*-MOE prevents activity of the unmodified strand.^84^ Cleavage of the passenger strand occurs between bases 9 and 10, suggesting that the modification prevents nuclease activity, thus inhibiting maturation of the pre-RISC. As expected, AGO2 loading assays reveal that the site-specific modification simultaneously increases and reduces uptake of the modified and unmodified strand, respectively. This same study reported further improved strand specific activity with an siRNA which combines the 2′-*O*-MOE at AS:9 and AS:10 with a 2′-*O*-Me at SS:14.

The substitution of the 2′-OH with a fluorine increases Watson–Crick pairing due to augmented enthalpy^85^ and in part by stabilization of the C3′-*endo* conformation.^83^ It is well tolerated in either strand; however, its use at the 5′ terminal had been preferred in the sense over the antisense strand.^41,51,86,87^ Formulations with alternating 2′-F and 2′-*O*-Me perform well,^41^ but reducing the number of 2′-F and detecting their ideal placement has led to the preferred design of Alnylam's advanced enhanced stabilization chemistry siRNA.^88^ Importantly, this design not only improved potency and duration of effect but also eliminated sense strand loading. While hepatotoxicity is a concern with siRNA drugs, studies with 2′-F and 2′-*O*-Me modified siRNA indicated that they are not the driving force behind murine hepatotoxicity of GalNAc-siRNAs.^89^

SiRNAs with 2′-deoxy nucleotides with a *S* or *R* configuration of a methyl on the 2′ carbon (Fig. 4) were evaluated for their influence on strand specific activity.^90^ These modifications were tested at positions AS:5, AS:6 AS:20 and SS:20. Modification of position AS:20 with either *R* or *S* configuration was found to improve the IC_50_. Interestingly, despite reduced potency, these modifications provided the most improved strand specific activity when placed at AS:5 or AS:6. Strand uptake assays were not performed but it is likely that effects are due to altered uptake.

### Non-ribose sugar modifications

Altritol nucleic acids (ANA) (Fig. 4) have a six membered sugar structure with a nucleobase bound to the 2′-(*S*)-position, a 3′-hydroxyl, and are linked to the backbone through the 4′ and 6′ carbons. Recently, the ANA modification was positionally screened on the antisense strand, which generally led to small losses in activity.^86^ Notably, modification of AS:2 was the most detrimental on activity and AS:16 had a negligible impact on activity. Modification of the sense strand decreased siRNA activity, suggesting low potential for strand selection. Furthermore, placement of an ANA at AS:6 or AS:7 was unable to significantly improve protein silencing.

The addition of a locked nucleic acid (LNA) (Fig. 4) to the 3′ termini of siRNA ends is well tolerated and provides thermal stabilization; however, only the sense strand will tolerate a 5′ LNA.^91,92^ An approximately 7.5 fold decrease in activity is observed when the LNA is placed at AS:1, and strand activity assays indicate dramatically reduced sense strand activity when the LNA is placed at SS:1.^92^ Elmén and colleagues also reported that poor strand selection can be improved through the addition of an LNA at SS:1. Notably, too many sense strand modifications tend to reduce antisense activity.^93^ This study also reported good activity of siRNA that carried LNAs at all termini except AS:1 in murine models. The toxicity of LNAs has been a concern for therapeutics, but the number and location of LNAs play a large role on the degree or presence of toxicity.^94^

The unlocked nucleic acid (UNA) (Fig. 4) has an open and flexible structure that results in thermal destabilization. Laursen and colleagues found that 3′ UNAs can reverse the thermodynamic stabilizing effect of 5′ modifications (such as LNAs), thus improving control over thermodynamic dependent strand selection.^95^ An UNA at the 5′ end prevents phosphorylation by Clp1 kinase, thereby abrogating binding to AGO2.^42^ This discovery agrees with other studies where UNA modifications inhibit activity of the corresponding strand.^14,96^ Chemical phosphorylation of a 5′ UNA modified strand recovered its activity and would likely benefit from a stabilized phosphate mimic such as 5′-*E*-VP. Importantly, this study also identified that an UNA at AS:11 had a dramatic deactivating effect, suggesting that it may be effective in the sense strand. The UNA was also shown to mediate miRNA-like off target effects when placed at AS:7.^97^ These approaches were combined in the development of an siRNA with several UNA modifications at select positions to reduce off-target events by over 10 fold compared to unmodified siRNA.^14^ A similarly flexible modification, the glycol nucleic acid (GNA) (Fig. 4) modification, is also not tolerated at positions 1 or 2 on antisense strand^98^ and may provide similar advantages as those identified with the UNA.

A guanidinocarbohydrazidomethyl-5-methyl uridine (GMU) (Fig. 4) modification was recently developed and placed within 3′ end of strands.^99^ When placed at the last two nucleotides of both strands, a 4.9-fold improvement in IC_50_ over the unmodified strand was reported. However, this value was based on a dilution series that was unexplainably unable to detect activity for one of three concentrations tested. Surprisingly, no significant knockdown was reported for an siRNA in which three nucleotides were modified (siRNA5), while similar designs with one, two, or four GMU modifications produced 50% to 95% knockdown. To explain these results, the authors proposed strand recruitment assays to measure strand uptake in AGO2; however, the methods reported measurements from whole cell, rather than AGO2-bound, RNA. If performed as described, these results indicate the absence of an antisense strand for siRNA5 in the cell, suggesting that no antisense strand was present for the siRNA in question. Therefore, the reported 4.9-fold improvement in siRNA carrying four GMU modification and reported 234-fold enhanced sense strand selection for siRNA5 warrants confirmation.

### Whole nucleotide substitutions

Nucleotide substitutions can add novel functionality to siRNA molecules by replacing one or more nucleotides with a structurally unique moiety. The size of individual substitutions/linkers can vary greatly but are best tolerated when they mimic the distance of natural nucleotides at approximately 3.4 Å per nucleotide. If the linker is placed in the antisense strand, it must not significantly prevent AGO2–RNA interactions.

A propargyl linker (Fig. 4) allows for the convenient addition of conjugates *via* click chemistry. The propargyl handle was introduced at SS:10 or SS:20 where it was used to add a folic acid moiety.^60^ Cancerous cells that overexpress the folate receptor, such as HeLa cells, mediate the uptake of the folic acid conjugated siRNA, making the siRNA specific for these cancerous cells. When the folic acid conjugate was placed at SS:20, strand selection was dramatically improved, which was not observed for the propargyl handle alone.^100^ This design differs from traditional folic acid conjugates which are bound by an alkyl, triethylene glycol, or polyethylene glycol linker,^34,57,58^ by holding the folic acid proximal. Understanding whether the structure/hydrogen bonding interactions, or steric bulk, of the folic acid moiety are responsible for the improved strand selection may help the development of 3′ conjugated siRNA.

Another modification that impacts strand selection is the azobenzene linker (Fig. 4), which photoisomerizes to the hydrophilic *cis* or hydrophobic *trans* isomer upon irradiation by ultraviolet (340–380 nm) or visible light (420–490), respectively.^101^ The length of azobenzene ends in *trans* form is 9.0 Å, while the *cis* form is shortened to 5.5 Å^102^ which disrupts the siRNA structure and prevents uptake by the RISC.^103–105^ Once delivered to cells, the inactive *cis*-form siRNA can be activated by light to control the rate of siRNA uptake by AGO2. Although azobenzene-modified siRNAs are less potent, improved strand selection is observed when placed at SS:8 or SS:10.^100^ Improved maximal knockdown was also reported, likely due to reduced sense strand uptake and increasing the AGO2 availability.

Replacing a nucleotide with a *p*-cyanostilbene linker (Fig. 4) facilitated the photocrosslinking of strands in siRNA.^106^ The *p*-cyanostilbene modification was appended on to the ends of a 24 bp siRNA, forming an overall length of approximately 26 bp. Notably, the unmodified 24 bp siRNA exhibited much higher sense than antisense strand activity. The 26 bp dsRNA, both before and after crosslinking, reduced sense strand activity to be comparable to the antisense strand. When the pre-crosslinked *p*-cyanostilbene was placed at AS:18, with a complementary *p*-cyanostilbene on the sense strand, dramatically improved strand selection and potency was observed. Once crosslinked, potency of the treatment substantially decreased. Similar experiments with the *p*-cyanostilbene placed at AS:14 were not effective.

### Alternative siRNA structure

**Fig. 5:**
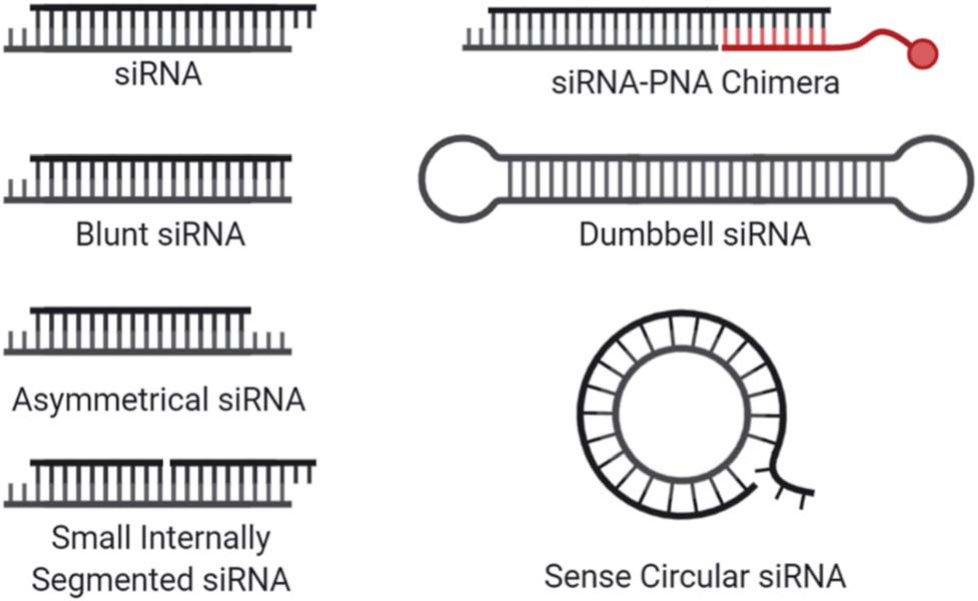
Structural designs used for strand selection in siRNA.

Blunt siRNAs (Fig. 5), which carry the typical 2 nt 3′ overhang on the antisense but not on the sense strand, preferentially load the antisense strand in the RISC, improving antisense activity while dramatically reducing sense strand activity.^107^ Further truncating the sense strand to create asymmetric siRNA consisting of a 19 nt long antisense strand and 16 nt long sense strand also provided good antisense activity while abolishing sense strand activity.^108^ Interestingly, while strand activity seems to be improved by these strategies, the sense strand is likely still recruited by AGO2, as microarray analysis indicated that miRNA-like off target effects may not be influenced by strand shortening.^109^ Strand recruitment analysis for these approaches would benefit our mechanistic understanding of reduced activity.

An siRNA molecule that carries a centrally cleaved sense strand, known as a small internally segmented interfering RNA (sisiRNA) (Fig. 5), prevent activity from the sense strand.^110^ Due to the short strand length, sisiRNAs require thermodynamically stabilizing modifications such as LNAs to prevent premature strand separation. Notably, these modifications are not correlated with increased antisense strand potency.^111^

Dumbbell-shaped nanocircular siRNA (Fig. 5), which carry a hairpin on either end, have been explored as Dicer substrates which require processing for uptake into the RISC.^112^ The addition of processing steps reduces the rate of RISC loading/saturation, but also complicates specificity concerns as several siRNA products are possible. A more specific approach to nanocircular siRNA was accomplished through the circularization of the sense strand which abolished sense strand knockdown.^113^ Based on competition assays, the thermodynamic stability of the circular duplex is approximately 17-fold lower than that of the corresponding linear siRNA duplex. A 21-mer circular sense strand was hybridized with 21-mer, 24-mer, or 27-mer antisense strand to identify ideal lengths. The 24-mer was most active, for which the authors suggest that more effective mRNA binding may occur due to a 24-mer RISC-guide complex, compared to a 21-mer. The promise of sense nanocircular siRNA warrants further refinement with alternative strand lengths and incorporation of chemical modifications, particularly those with thermodynamically stabilizing properties.

Recently, siRNA-peptide nucleic acid (PNA) chimeras were used to non-covalently bind a conjugate through the hybridization of a PNA to asymmetrical siRNA (Fig. 5).^114^ The preferred design for this approach was a 21-mer antisense strand, hybridized to the 5′ end of a 30-mer sense strand, where the overhanging nucleotides on the sense strand was used for the non-covalent attachment of an 9-mer peptide nucleic acid (PNA) bound to a conjugate. This simplifies the conjugation process and was successfully incorporated into a liver-targeting nanocomplex.^115^ The length of the sense strand likely prevents significant activity, but strand selection assays are needed to properly evaluate the therapeutic risk of the sense strand and whether chemical modifications may provide additional value.

## Conclusions

**Fig. 6:**
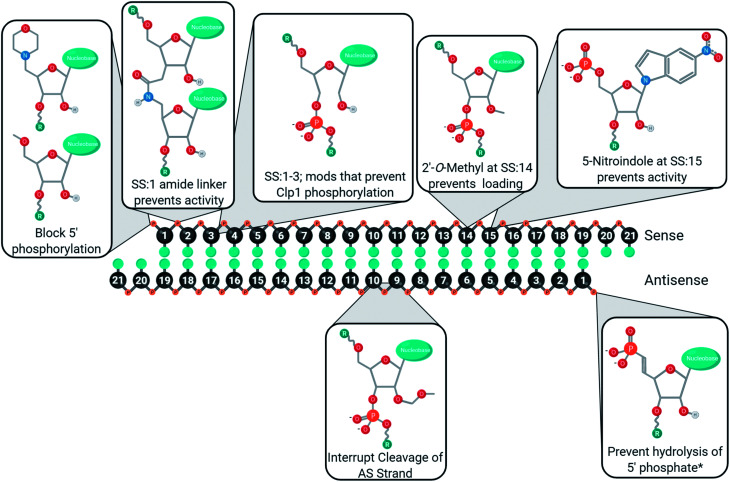
Strategies used to mitigate sense strand of uptake into RISC. Note that utility of modifications outside of their tested targets/tissues is unknown. *Beneficial for heavily modified siRNAs as the kinase Clp1 may not efficiently phosphorylate modified 5′ ends.

Chemical modifications are used in siRNA for managing cell delivery, potency, nuclease resistance, immunogenicity, and off-target gene silencing. The complexity of these demands requires a variety of modifications, and rarely will one modification not have an impact on other chemical characteristics. For example, modifications of the 5′ antisense end often prevent phosphorylation, leading to poor activity unless a stabilized phosphate mimic is used. As such, siRNA design requires careful consideration of which modifications will be used, how many nucleotides will be modified, and where the modifications will be placed. Furthermore, many of the chemical modifications discussed in this review have been investigated in different laboratories using unique sequences, gene targets, cell lines, and/or organisms. As such, these figures represent a convenient guide, rather than a perfected catalogue, to the development of new modifications or siRNA designs. Many positions, for better or worse, impact off-target gene silencing through the modulation of strand selection during RISC maturation. To aid in the development of siRNAs with minimal sense strand selection, the currently successful chemical strategies for improving strand selection are summarized in Fig. 6. Improving strand selection is primarily achieved through the control of the 5′ phosphorylation status, altering internal structure to prevent cleavage of an antisense loaded in the passenger position, or altering siRNA structure that prevents sense strand capture. Improving siRNA designs with these strand selection modifications will support the development of effective, safe, and potent therapeutics.

## Conflicts of interest

There are no conflicts to declare.

## Supplementary Material
